# A TD-DFT-Based Study on the Attack of the OH· Radical on a Guanine Nucleotide

**DOI:** 10.3390/ijms231710007

**Published:** 2022-09-02

**Authors:** João Santiago, Jhaison C. de Faria, Miguel San-Miguel, Mario A. Bernal

**Affiliations:** 1 Instituto de Física Gleb Wataghin, Universidade Estadual de Campinas, Campinas 13083-859, SP, Brazil; 2 Instituto de Química, Universidade Estadual de Campinas, Campinas 13083-970, SP, Brazil

**Keywords:** chemical attack, hydroxyl, DNA, TD-DFT

## Abstract

Heavy charged particles induce severe damage in DNA, which is a radiobiological advantage when treating radioresistant tumors. However, these particles can also induce cancer in humans exposed to them, such as astronauts in space missions. This damage can be directly induced by the radiation or indirectly by the attack of free radicals mainly produced by water radiolysis. We previously studied the impact of a proton on a DNA base pair, using the Time Dependent-Density Functional Theory (TD-DFT). In this work, we go a step further and study the attack of the OH· radical on the Guanine nucleotide to unveil how this molecule subsequently dissociates. The OH· attack on the H1′, H2′, H3′, and H5′ atoms in the guanine was investigated using the Ehrenfest dynamics within the TD-DFT framework. In all cases, the hydrogen abstraction succeeded, and the subsequent base pair dissociation was observed. The DNA dissociates in three major fragments: the phosphate group, the deoxyribose sugar, and the nitrogenous base, with slight differences, no matter which hydrogen atom was attacked. Hydrogen abstraction occurs at about 6 fs, and the nucleotide dissociation at about 100 fs, which agrees with our previous result for the direct proton impact on the DNA. These calculations may be a reference for adjusting reactive force fields so that more complex DNA structures can be studied using classical molecular dynamics, including both direct and indirect DNA damage.

## 1. Introduction

Ionizing particles induce biological damage by interacting with DNA. Although this principle has been used for a long time, e.g., for treating cancer in radiotherapy, there is not yet a full understanding of the physicochemical mechanisms involved in the early stage of the DNA damage induced by radiation. This matter is of great importance for applications in the medical and aerospace fields.

DNA damage induction by ionizing particles is a complex process that occurs both by the direct impact of ionizing particles or indirectly by the action of free radicals. These chemical species are mainly created by water radiolysis [1]. The hydroxyl radical (OH·) is the most abundant species generated by water radiolysis and is very reactive. It can produce hydrogen abstraction from the nucleotide pair. Given that the HO–H bond dissociation energy is 5.20 eV, in contrast, for C–H bonds, it is only 3.91–4.26 eV [2]. Experimental studies have shown that there is an order of reactivity with respect to the possible hydrogen abstraction sites in deoxyribose [3].

The Monte Carlo (MC) method has been used over the last few decades to simulate the early DNA damage [4,5,6]. This approach includes not only the simulation of radiation transport, but also the production and transport of chemical species generated by water radiolysis [7]. In short, particle and chemical species tracks are superimposed onto a DNA geometrical model, and DNA damage probabilities are determined. The problem is that models for linking this interaction process to effective DNA damage are still very simplistic. To tackle this problem, we are using the TD-DFT methodology to better understand how the DNA molecule is damaged by the impact of heavy charged particles. In a previous work [8], we studied the dissociation of a DNA base pair after the impact of a proton, using the TD-DFT implemented in the Octopus code [9,10]. This approach should shed some light on the DNA damage induced by ionizing radiation. For instance, the energy required for a strand break could be found. This parameter is fundamental for MC-based methodology for studying the early DNA damage. Furthermore, we need an understanding of the chemical or indirect damage to the DNA after irradiation with ionizing particles. Sadr et al. [11,12,13,14] have used the DFT to study the fragmentation of cytosine, adenine, guanine, and uracil bases. Okutsu et al. [15,16] investigated the attacking mechanisms of radicals to base pairs in water, and Santosh and Abolfath [17], the relation of the molecule charge with the bond dissociation and fragmentation behavior of DNA bases. However, no time-dependent calculation was carried out by these authors, nor did they study the dynamics of the collision. In addition, classical molecular dynamics simulations based on Reax force fields have also been used to study this problem, both for the the direct damage [18] and the chemical one [19], yet without the explicit description of the proton-DNA collision process.

In the present work, we go a step further and study the dynamical evolution of a nucleotide after the attack of an OH· free radical. The TD-DFT formalism, implemented in the Octopus code, was used to simulate the H abstraction by the OH· radical. Specifically, H atoms from the sugar-phosphate group were used as targets for the OH· radical, since their abstraction may lead to strand breaks. It is also worth noting that abstraction cannot be the most probable process for vinylic hydrogens that are more tightly bound. In those cases, the OH· radical is capable of additional reactions, for example on the C_4_, C_5_, and C_8_ atoms of guanine nucleotide, which are not investigated here but may have a relevant role depending on the kinetics of the system [20]. Due to the high reactivity of the hydroxyl, a large number of products can be detected [21,22]. Given this background, the present work specifically studied the reactions of the hydroxyl with the guanine nucleotide, specifically in deoxyribose, and the resulting perturbation in the whole nucleotide. It should be remarked that this work not only aims at understanding the process in question but also at creating a reference based on the TD-DFT for further adjusting reactive force fields used in classical dynamics calculations. Classical calculations demand much fewer computing resources, so more complex systems can be studied.

## 2. Results

**Figure 1:**
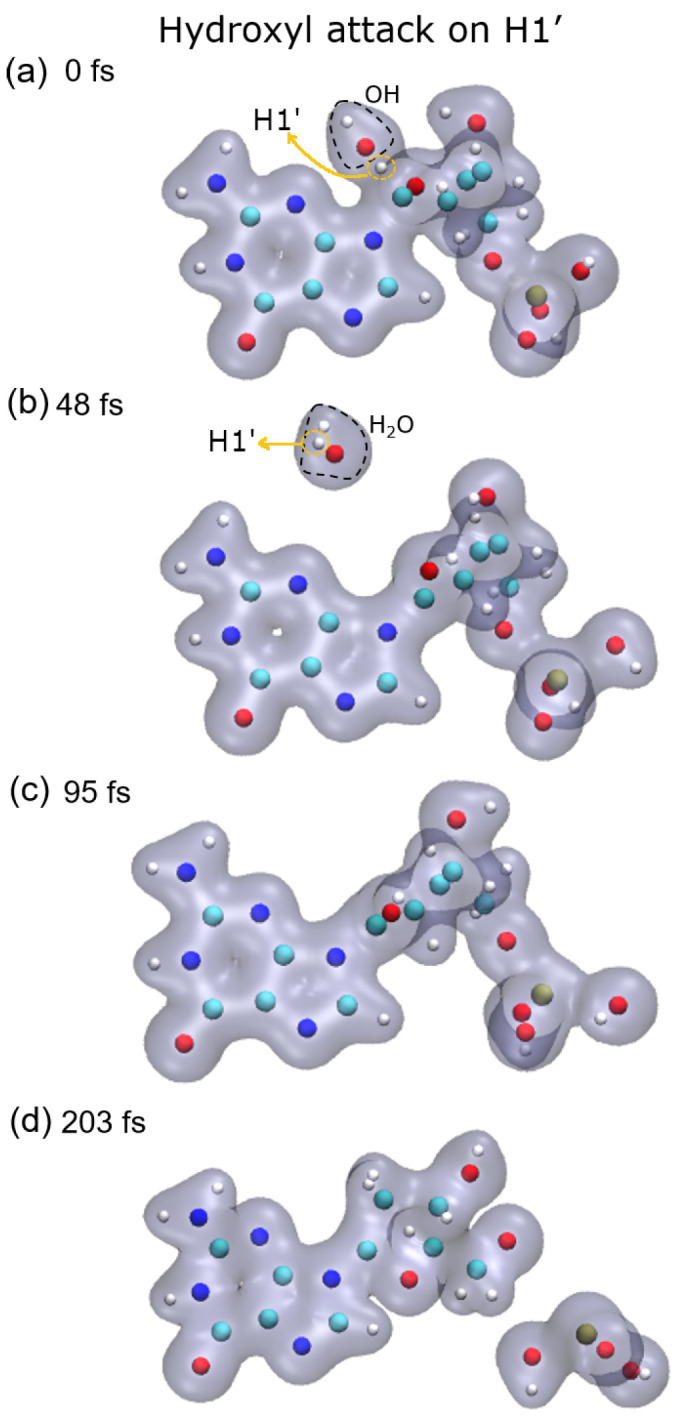
Time evolution of the case of hydrogen H1′ as target: (**a**) initial instant with the hydroxyl close to the target; (**b**) hydroxyl already performed the abstraction forming water that moves away from the rest of the molecule; (**c**) molecule oscillating in the middle of the dissociation process; (**d**) phosphate group dissociates from the base and sugar, which remain together. Atoms, H: white, C: cyan, O: red, N: blue, P: ochre.

**Figure 2:**
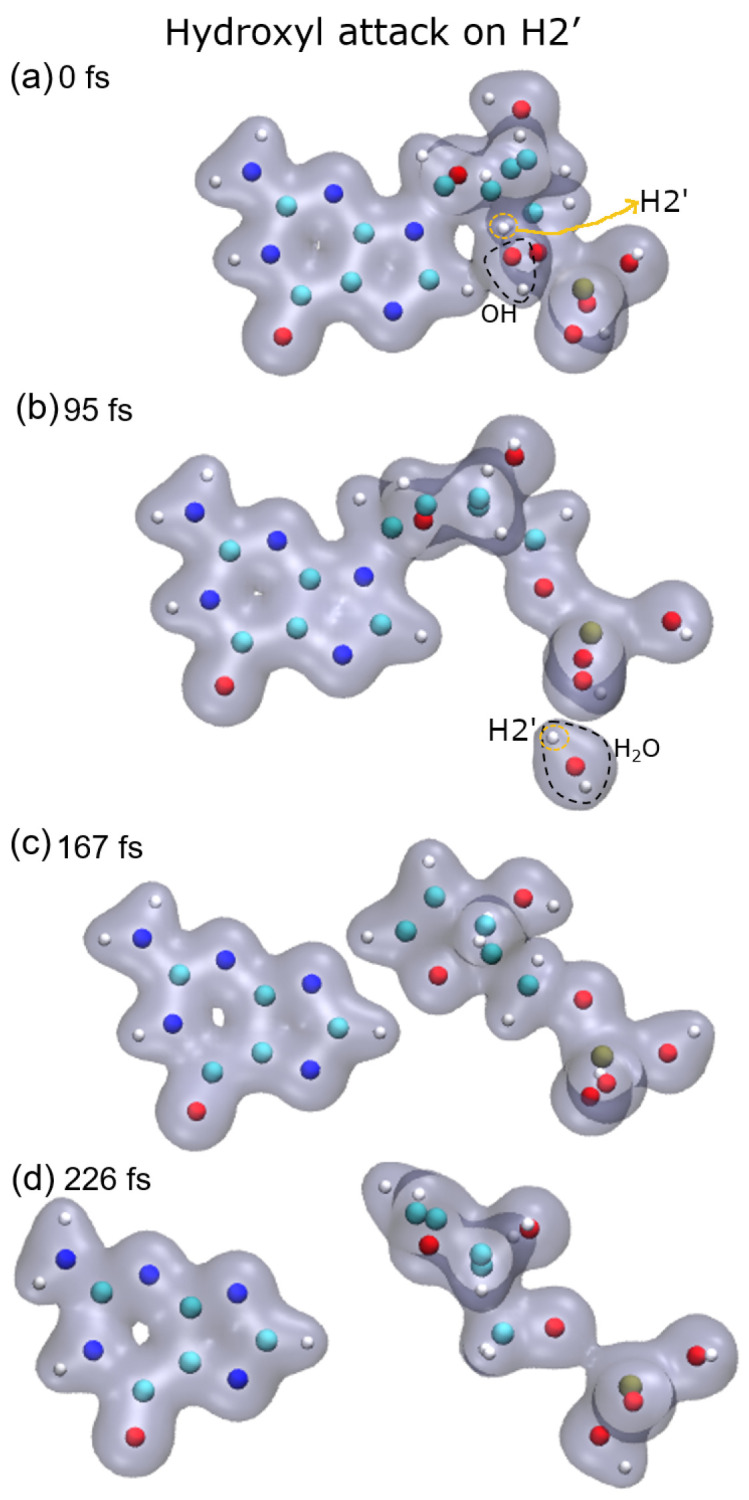
Time evolution of the case of hydrogen H2′ as target: (**a**) initial instant with the hydroxyl close to the target; (**b**) hydroxyl already performed the abstraction forming water that moves away from the rest of the molecule; (**c**) deoxyribose dissociates from the base; (**d**) phosphate group dissociates from deoxyribose. Atoms, H: white, C: cyan, O: red, N: blue, P: ochre.

**Figure 3:**
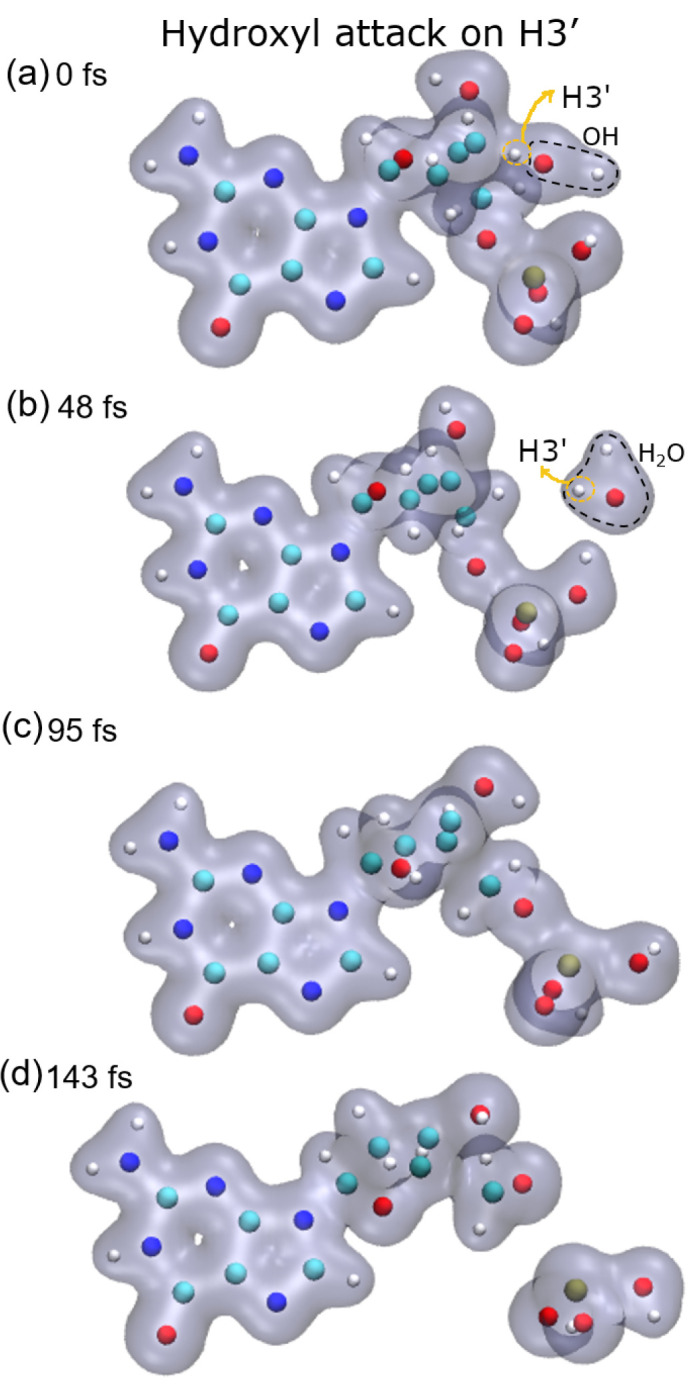
Time evolution of the case of H3′ as target: (**a**) initial instant with the hydroxyl close to the target; (**b**) hydroxyl already performed the abstraction forming water that moves away from the rest of the molecule; (**c**) molecule oscillating in the middle of the dissociation process; (**d**) phosphate group and fragment containing C5′,O5′ and hydrogen, dissociates from deoxyribose. Atoms, H: white, C: cyan, O: red, N: blue, P: ochre.

**Figure 4:**
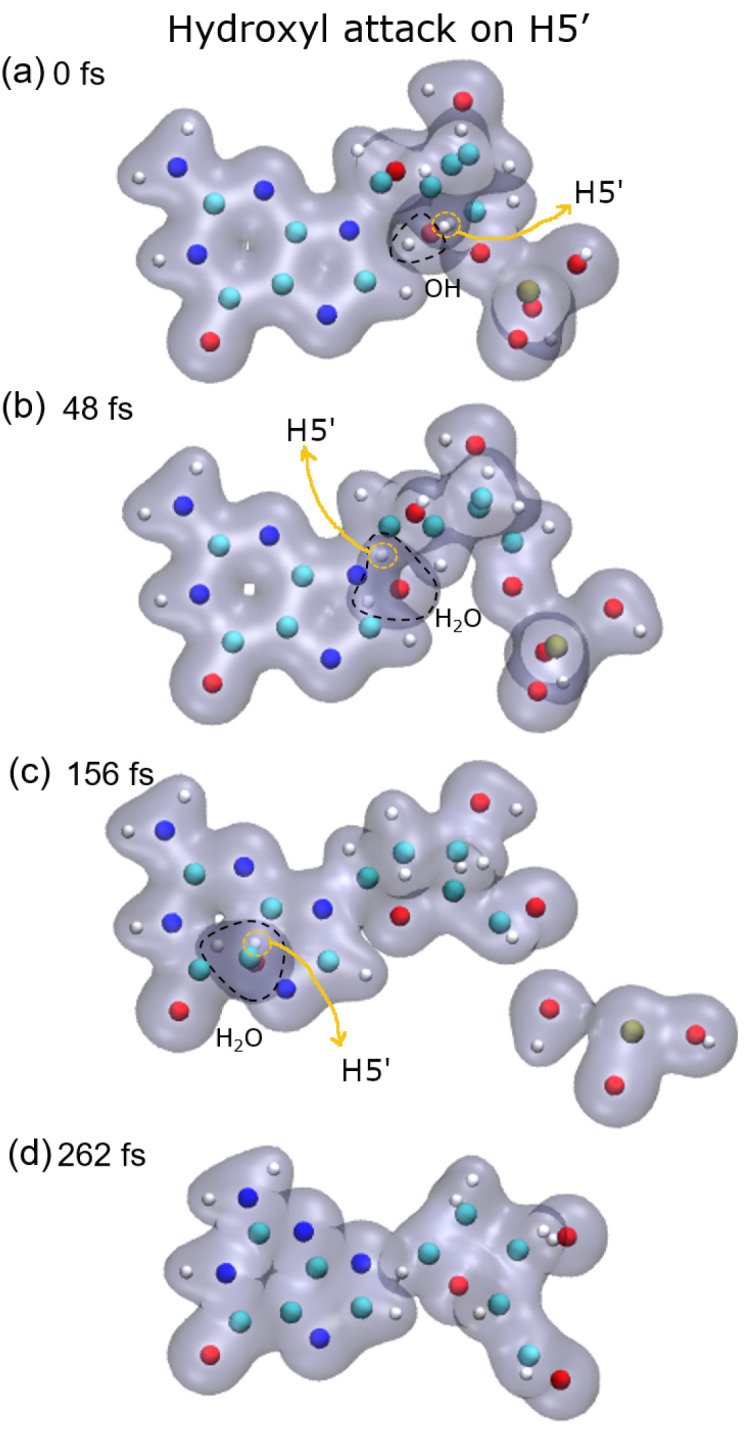
Time evolution of the case of H5′ as target: (**a**) initial instant with the hydroxyl close to the target; (**b**) hydroxyl already performed the abstraction forming water that moves away from the rest of the molecule; (**c**) phosphate group dissociates from the base, and it can be seen that it fragments into a free hydroxyl radical and a phosphate cluster. (**d**) deoxyribose dissociates from the base. Atoms, H: white, C: cyan, O: red, N: blue, P: ochre.

Figure 1, Figure 2, Figure 3 and Figure 4 show snapshots of the evolution of the nucleotide after the attack of the hydroxyl radical on the H target atoms considered in this work. Calculations were made up to about 250 fs. The gray shaded areas represent 0.04 electronic density isosurfaces. Typically, the target hydrogen atom is first repelled towards the carbon by the hydroxyl oxygen, reaching minimum distance at about 3 fs. Later, the hydrogen atom pulls back closer to the oxygen, thus forming the O–H bond, while the C–H bond dissociates after reaching the bond distance of 1.09 a.u at about 6 fs. Then, the formed water molecule continues to move away from the guanine base, having a velocity of about 0.002 a.u. at 10 fs, st a distance of 4.8 a.u. from the carbon from which the hydrogen atom was abstracted.

**Figure 5:**
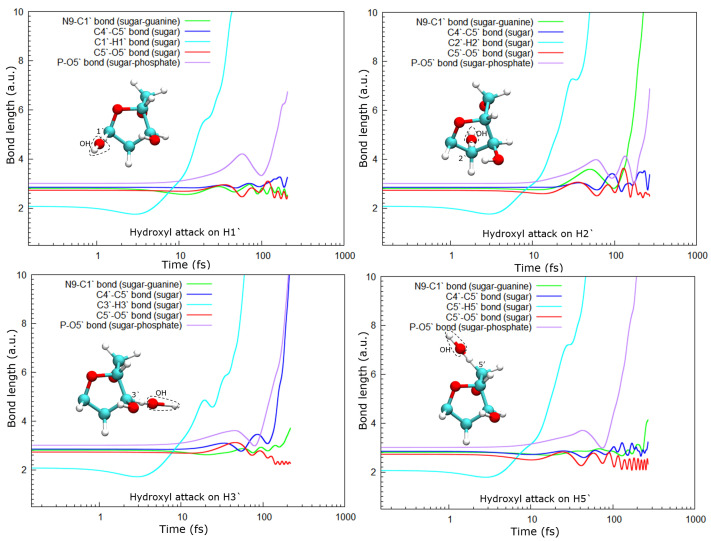
Some important bond lengths as a function of the time for different targets. Only the sugar moiety is shown here to better show the attack points. Atoms, H: white, C: cyan, O: red.

Figure 5 shows the lengths of some important bonds as a function of time after the attack of the hydroxyl group on the hydrogen atoms. Thus, we can determine which bond dissociates after the hydrogen abstraction and what the corresponding dissociation time scale is. It can be observed that all hydrogen targets (cyan lines) dissociate from the corresponding C atom following a very similar temporal trajectory. Initially, there is a compression of the C–H bond due to repulsion from the OH· radical, and then the H atom pulls back and binds to the radical, forming water and leaving the site. The typical dissociation time is about 5 fs, after the effective approximation of the OH· radical. Some fluctuations observed at longer times can be attributed to water molecule spinning.

## 3. Discussion

As a consequence of the hydrogen abstraction, the entire molecule is strongly perturbed and, after some time, dissociates. Dissociation products and times depend on the site from which the hydrogen was abstracted. For instance, the abstraction of H1′ leads to the dissociation of the phosphate group from the sugar moiety by breaking the P–O5′ bond, while O5′ remains bound to the C5′ of the deoxyribose (see Figure 5, top-left panel).

In the case of the radical attack on H2′ (see Figure 5, top-right panel), the N9–C1 bond is broken before the P–O5′ one. Thus, in this case, three fragments are produced, namely the guanine base, the deoxyribose, and the PO_3_H_2_ group (see Figure 2). As shown in our previous work [8], these three groups are very stable and are the most probable fragments after the dissociation of a DNA base pair in a vacuum. However, after the attack on H3′, we observed the breaking of the C4′–C5′ bond, besides that of the P–O5′ bond, forming the fragment COH_2_, while the sugar remains attached to the base.

Lastly, after the abstraction of H5′ (see Figure 5, bottom-right panel), it can be seen that the sugar remains bonded to the base for a long time, about 150 fs and then dissociates. Meanwhile, the P–O5′ bond breaks well before, nearly half the time, and a PO_2_ fragment and a free hydroxyl radical are produced in this case.

In all cases, the C5–O5′ bond length oscillates around a new length, shorter than the one in the original molecule. This could be a consequence of the detachment of the phosphate group from the sugar. At least up to the time studied here, this bond does not break in any of the cases accounted for in this work.

The stability of DNA depends on several factors, including the DNA structure, solvation conditions, and the presence of a counter-ion and other molecules such as proteins. Recently, Nieuwland et al. have shown, using DFT calculations, that the conformation of the DNA double helix is also sequence-dependent [23]. They showed that charged DNA is less stable than that neutralized since the latter reduces Coulomb spurious interactions. In our calculations, only proton-neutralized DNA has also been considered because it is the most stable conformation of an isolated nucleotide. Our results show that hydrogen abstraction by the OH· is an ultra-fast process, taking place in up to a few femtoseconds. After removing the hydrogen atoms studied, the guanine nucleotide is left in an excited state that decays by dissociation, where the P–O5′ bond is always broken. However, the C4′–C5′ bond only dissociates when the H3′ atom is removed, at least up to the maximum time used in our calculations of about 250 fs. The N9–C1 bond that binds the sugar and the base is broken after the abstraction of H2′, H3′, and H5′, while removing H1′ does not seem to lead to the sugar-base dissociation. This is somehow curious since H1′ is the closest hydrogen atom to the N9–C1 bond among those studied, and its abstraction does not produce the dissociation of this bond.

In our former work [8], we studied the dissociation of a nucleotide pair after the impact of a 4 keV proton. Two collision setups were used, one with the proton impacting at the center of the DNA base pair, along the axis normal to the pair plane, and the other with the proton approaching one of the phosphorous atoms at zero impact parameter. In the first collision setup, the momentum transfer is relatively low, and the dissociation time scale is similar to that obtained in the current work, that is, after hydrogen abstraction, about 100 fs. However, for the proton–phosphorus head on collision, the momentum transfer is about four times higher than in the central impact, which leads to 10-fold faster dissociation of the phosphate group. An important difference in the dissociation pattern between the proton impact case and the hydrogen abstraction is that during the latter, large amplitude oscillations of the broken bond lengths are observed before the dissociation.

## 4. Materials and Methods

### 4.1. Theoretical Background of the TD-DFT

Solving Schrödinger equations system for multielectronic systems is a very hard task, mainly due to the difficulty involved in describing interaction potential between electrons, which is also responsible for coupling equations in the resolution of the Schrödinger equations. Kohn and Sham developed the density functional theory, introducing the exchange-correlation potentials which account for such interactions. Therefore, the system of equations can be decomposed into decoupled equations that can be solved independently, yet in a self-consistent fashion. According to this formalism, we can solve the electronic wave equation for the *i*-th Kohn–Sham orbital

(1) $$\begin{array}{c} \left\{-\frac{1}{2}\nabla^{2}+v_{eff}\left[n\right]\left(r\right)\right\}\phi_{i}\left(r\right)=E_{i}\phi_{i}\left(r\right) \end{array}$$

The first term is the kinetic energy, and the second one, the effective potential, given by the following terms:

(2) $$\begin{array}{c} v_{eff}\left[n\right]\left(r\right)=v_{Hartree}\left[n\right]\left(r\right)+v_{ext}\left(r\right)+v_{xc}\left[n\right]\left(r\right). \end{array}$$

The first term is the Hartree potential, given by

(3) $$\begin{array}{c} v_{Hartree}\left[n\right]\left(r\right)=\int \frac{n(r^{\prime })}{|r-r^{\prime }|}d^{3}r^{\prime }. \end{array}$$

The second one is the external potential $v_{ext}\left(r\right)$ that, in the absence of electromagnetic fields, is given by the interaction of the electron with all the *M* nuclei of the system

(4) $$\begin{array}{c} v_{ext}\left(r\right)=-\sum_{k=1}^{M}\frac{Z_{K}}{\left|r-R_{K}\right|}, \end{array}$$

where $Z_{K}$ and $R_{K}$ are the charge and position of the *K*-th nucleus.

The last term is the so-called exchange-correlation potential that contains all the many-body information of the coupled electronic system.

Both the Hartree and exchange-correlation potentials are functionals of the electronic density, defined by:

(5) $$\begin{array}{c} n\left(r\right)=\sum_{i=1}^{N}{\left|\phi_{i}\left(r\right)\;\right|}^{2} \end{array}$$

Thus, guaranteed by the Hosenberg–Kohn theorem that the ground-state density n(r) uniquely determines the potential, the orbital Equation (1) can be solved in a self-consistent way, obtaining the electronic density of the fictitious non-interacting system, which matches the interacting one.

Later, with the extension of the fundamental theorems of DFT by Runge and Gross, we can also write the time-dependent equations of Kohn–Sham orbitals as:

(6) $$\begin{array}{c} \left\{-\frac{1}{2}\nabla^{2}+v_{eff}\left[n\right]\left(r,t\right)\right\}\phi_{i}\left(r\right)=i\frac{\partial }{\partial t}\phi_{i}\left(r,t\right) \end{array}$$

In this way, the electron density for each time step is obtained in a similar way to the ground-state calculation. Nuclei are treated classically, moving under the influence of the field induced by the electrons and nuclei. This treatment is known as the Ehrenfest dynamics and can be described by the following equations

(7) $$\begin{array}{ccc} i\hbar \frac{\partial \Phi (r,R,t)}{\partial t} & = & {\hat{H}}_{el}\left(r,R\right)\Phi \left(r,R,t\right) \end{array}$$

(8) $$\begin{array}{ccc} M_{K}{\ddot{R}}_{l} & = & -\nabla_{K}<{\hat{H}}_{el}\left(r,R\right)>, \end{array}$$

where ${\hat{H}}_{el}$ is the electronic Hamiltonian of the system, which includes the potentials shown in Equations (3) and (4); Φ(r,R,t) is the total electronic wave function that can be expanded onto the $\phi_{i}$ Kohn–Sham basis. In this equation, $M_{K}$ is the mass of the *K*-th nucleus. According to this approach, nuclei are displaced at each time step; then the new density distribution is found. Under this further electronic distribution, nuclei are allowed to move, and so on. Details on this formalism can be found elsewhere [24].

It is worth noting that inner shell electrons can be coupled to the nucleus, and a pseudopotential is assigned to this cluster that interacts with the rest of the electrons. In this way, the number of orbitals is reduced, and so is the computation time.

### 4.2. System Setup

**Figure 6:**
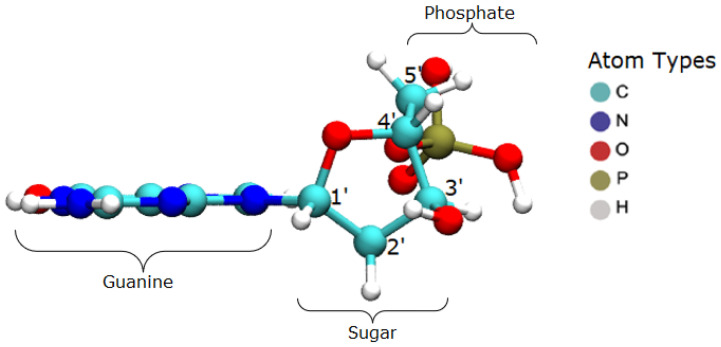
Guanine base setup.

The target system consists of the guanine base in the B-DNA configuration, at rest, as visualized in Figure 6. The nucleotide has the whole phosphate group, starting with the atom O3′ and continuing to the next O3′, which would belong to the neighbor base. These terminal oxygen atoms were kept fixed in order to emulate the binding effect with the adjacent bases.

The hydroxyl was positioned in four different configurations, having a thermal velocity corresponding to T = 293 K, in order to abstract target hydrogen atoms from the deoxyribose, namely atoms H1′, H2′, H3′, and H5′. In each case, the hydroxyl group was placed at a distance of 1.5 a.u. from the target hydrogen, aligned with the C–H bond of the hydrogen. Placing the hydroxyl radical not too far from the target hydrogen atom led to computing time-saving.

### 4.3. Ground State Calculation

The Octopus code version 10.5 was used for the calculations using the TD-DFT formalism. First, a ground-state calculation was performed for the DNA + OH· system. The exchange and correlation functionals were the LDA and the modified Perdew–Zunger LDA, respectively. The grid spacing used in the three Cartesian directions was 0.4 a.u., whereas the box in which the system was inserted has the dimensions of 30 × 20 × 20 a.u.${}^{3}$. These dimensions and grid spacing were chosen after the optimization of the system energy. In addition, a complex potential was used at the box boundaries in order to avoid electronic charge reflection in the box walls. With the use of pseudopotentials, the system has 133 valence electrons for 39 atoms, distributed in 67 orbitals.

### 4.4. Time-Dependent Calculations

After calculating the ground-state, the time evolution was performed using the AETRS (Approximately Enforced Time-Reversal Symmetry) propagator, with a constant time step of 0.05 a.u. (1.2 $\times \;10^{-3}$ fs). A velocity of 0.0003 a.u was imposed to the OH· radical along the C–H bond direction, equivalent to 293 K. Calculations were made up to about 200 fs. The rendering of the time evolution of the electronic system was performed using the VMD software [25].

## 5. Conclusions

The present TD-DFT study investigated the dynamics of the hydrogen abstraction from a nucleotide by the OH· free radical, including the subsequent dissociation of the target molecule, a guanine nucleotide. High computational costs are involved in this kind of calculation, even for a not-too-large molecule. This limited the total computing time to about 250 fs, but it was enough to observe the dissociation of the target molecule after hydrogen abstraction. In addition, a systematic study including the other three nitrogenous bases could not be performed in a reasonable time. The TD-DFT formalism, implemented in the Octopus code in conjunction with the Ehrenfest dynamics, shows to be a powerful tool for studying the hydrogen abstraction by this free radical and the nucleotide dissociation, which opens the possibility to studying the attack by other water radiolysis products with interest in radiobiology. This study also shows that hydrogen abstraction is an ultrafast process that leaves the nucleotide excited and then decays by dissociation. This dissociation is commonly preceded by large oscillations of the involved bond length and leads to the formation of three main fragments, the base, the sugar, and the H_2_PO_3_· group. Even with the limited number of calculations, studies like this can be useful for investigating and improving biophysical models on DNA damage induced by ionizing radiation, either in the scope of direct interactions or by action of free radicals, as this study. It can serve as a reference for adjusting classical molecular dynamics models, such as the REAX force-field. With an efficient classical model, computing times would be several orders of magnitude lower than in TD-DFT calculations, so more complex DNA structures could be studied.

## Data Availability

Not applicable.
